# A Framework for Bioinformatic Reporting in Prenatal Sequencing: Insights From a Systematic Review

**DOI:** 10.1002/pd.70085

**Published:** 2026-01-30

**Authors:** Ashley J. Pritchard, Karen Mei Xian Lim, Graeme Smith, Elizabeth Scotchman, Alexander Gibbs, Patrick Lombard, Natalie J. Chandler

**Affiliations:** ^1^ NHS North Thames Genomic Laboratory Hub Great Ormond Street Hospital for Children NHS Foundation Trust London UK; ^2^ Department of Obstetrics and Gynaecology National University Hospital Singapore; ^3^ Genetics and Genomic Medicine UCL Great Ormond Street Institute of Child Health London UK

## Abstract

Genomic sequencing has become a key tool in the investigation of foetal anomalies, with a growing shift from targeted panels to exome and genome sequencing. These broader approaches generate significantly more data, underscoring the need for robust bioinformatics pipelines. However, practices vary widely between laboratories. This systematic review explores current differences in bioinformatics workflows, the transparency of reporting, and the clinical impact of these variations. Using a search strategy from a previous review of prenatal sequencing studies (2018–2022), we identified 89 new records. Combined with 65 from the earlier review, a total of 154 articles were included. Data extraction focused on bioinformatics pipeline details across all analytical stages, with attention to clinical relevance. We found that reporting of bioinformatics methods was frequently incomplete. Tool names and versions were often omitted, quality control steps were poorly described, and filtering strategies lacked reproducibility. These deficiencies in reporting hinder readers from fully interpreting the sequencing results and understanding the potential limitations. To address this, we propose a checklist of essential bioinformatics metrics to improve reporting standards and support reproducible, clinically meaningful analyses.

## Summary

- What is already known about this topic?
  ◦ Bioinformatics pipelines for prenatal next generation sequencing data are well established.
- What does this review add?
  ◦ Bioinformatics strategies should be specifically tailored for prenatal and postnatal sequencing data, respectively.
  ◦ Strategies for reporting bioinformatics pipelines for transparency of methodological design.
  ◦ Potential clinical consequences of the current lack of transparency in methodology reporting and its impact on interpretation of results and limitations.

## 1 Introduction

In the last decade, the clinical utility of next generation sequencing for investigating foetal anomalies has been demonstrated [1, 2, 3]. The principles, rationale, and unique challenges of prenatal sequencing are also well established [4].

As sequencing costs have declined, prenatal sequencing studies have increased. This is reflected in the growing number of articles identified in literature reviews. In 2018, 14 articles were identified [4], in 2022, 66 papers were included [2], and 89 in 2025 (Lim *et a*l under review). There has also been a shift from targeted gene panels to broader approaches such as exome and genome sequencing. These methods generate substantially larger volumes of data, underscoring the need for well‐designed bioinformatics pipelines.

A clinical bioinformatics pipeline typically involves three stages: primary, secondary and tertiary analysis. Primary analysis converts raw sequencer signals to nucleotide bases; secondary aligns these sequence reads to a reference genome and calls variants at discordant sites; and tertiary annotates, filters, and prioritises variants to identify those most likely to be disease‐causing. Therefore, accurate variant detection depends on robust pipelines.

However, foetal sequencing pipelines vary widely and lack standardisation [5]. Stringent filtering improves efficiency by prioritising variants most likely to explain the foetal phenotype, reducing manual review time, but may miss diagnoses such as mosaicism or variants inherited from a seemingly unaffected parent [6]. More permissive filters reduce this risk but require a more manual curation of variants, thereby increasing the turn‐around time and costs. This review examines current differences in bioinformatics practices, the transparency of their methodologies and the clinical implications of these variations.

We reviewed the full text papers identified in the systematic review on prenatal sequencing by Mellis et al. [2] and repeated their search strategy to identify additional studies published between October 2021 and January 2025. Records from both searches were reviewed to extract data on bioinformatics strategies. Diagnostic yield and further sequencing details will be presented separately (Lim et al. under review).

## 2 Methods

### 2.1 Protocol and Registration

This review was prospectively registered on PROSPERO (reference CRD420251110938) on 28^th^ of August 2025. The same research protocol outlined by Mellis et al. [2] was applied to studies published between October 2021 and January 2025.

### 2.2 Information Sources and Search Strategy

In brief, electronic searches of MEDLINE, Embase, Cochrane library, and Web of Science were conducted for records published between October 2021 and January 2025. Search terms were variations on the keywords ‘prenatal diagnosis’ (e.g., ‘foetal’, ‘antenatal’) and ‘sequence analysis, DNA’ (e.g., ‘exome sequencing’, ‘genome sequencing’). Studies were included if they met the following criteria: (i) retrospective or prospective cohorts of 10 or more pregnancies undergoing sequencing for foetal structural anomalies, (ii) chromosomal analysis was non‐diagnostic, (iii) testing was initiated based on the prenatal phenotype, and (iv) full text report was available in English language. Records were excluded if they were reviews, commentaries, series of fewer than 10 cases, or sequencing studies focused solely on novel gene discovery. Titles and abstracts were screened by three independent reviewers. Full texts of potentially relevant abstracts were assessed against inclusion and exclusion criteria, with disagreements resolved through discussion.

The full strategy is detailed by Mellis et al. [2], and the updated sequencing review (Lim et al. under review).

### 2.3 Data Extraction

Bioinformatics pipeline data were extracted by one reviewer (KL) and checked by another (AP). Where available, this included: location, sample size, inclusion criteria, sequencing approach, technical details, quality control measures, and filtering and prioritisation strategies. As this review did not assess diagnostic yield, reported sample sizes differ from those in Mellis et al. [2]. In our review, sample size refers to all probands or trios sequenced, regardless of whether concurrent copy number variant analysis obtained a diagnosis which explained the phenotype.

### 2.4 Data Analysis

Data from all stages of the bioinformatics pipeline was analysed, with particular attention to missing or poorly reported fields that could have direct clinical implications.

## 3 Results

### 3.1 Study Cohort and Characteristics

**FIGURE 1:**
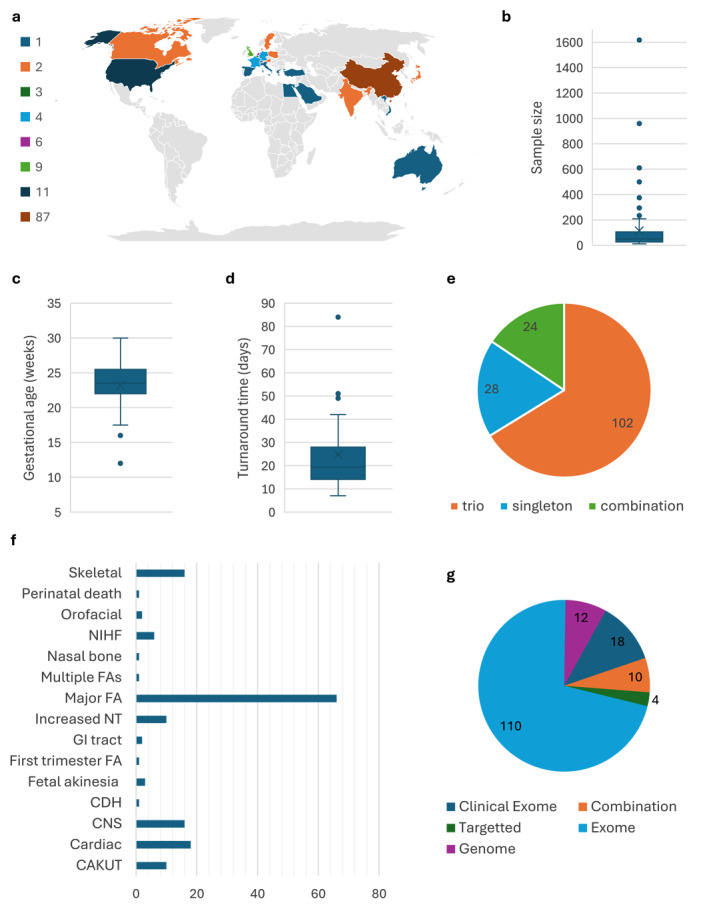
Summary of study characteristics across 154 prenatal publications (where reported). (a) Laboratory location by geographic region, (b) sample size distribution, (c) average gestational age (*n* = 123), (d) average turnaround time (*n* = 62), (e) proband versus family‐based testing and (f) number of studies per clinical indication. (g) sequencing approach. FA: foetal anomaly, CNS: central nervous system, CAKUT: congenital abnormality of the kidney or urinary tract, NT: nuchal translucency.

Database searches identified 5705 unique records. After title and abstract screening, 150 full texts were assessed for eligibility, with 89 included in the final review. These were combined with 72 records from the Mellis review, of which six were excluded due to shared bioinformatics pipelines. A further record was removed as it was a letter to the editor and thus not expected to contain bioinformatics data. In total, 154 articles describing prenatal sequencing for the investigation of structural anomalies identified by imaging, published across 55 journals and 25 countries (Figure 1a), were analysed.

Sample sizes reported ranged from 11 to 1618 foetuses (Figure 1b). DNA was obtained between 10 weeks' gestation and the postnatal period, following either delivery or termination of pregnancy, but interpretation of results only considered the prenatal phenotype (Figure 1c). Turnaround times were reported in 39.6% (61/154) of studies, ranging from 4 to 59 days (Figure 1d).

Trio analysis (parents and proband) was performed in 102 studies (66.2%), singleton analysis in 28 (18.2%) and a combination analysis in 24 (15.6%) (Figure 1e).

Overall, 44.8% (69/154) of studies were performed for the indication of a major foetal anomaly suggestive of a genetic cause. The remaining 85 studies were based on a specific phenotype (e.g., abnormal corpus callosum) or phenotypic group (e.g., central nervous system) (Figure 1f).

Exome sequencing was the most common approach (120/154, 77.9%), followed by clinical exome capture (19/154, 12.3%). Genome sequencing was used in 15 (9.7%) studies, mostly from 2022 onwards (11/15, 73.3%). Targeted gene panels were sequenced in seven studies (4.5%) (Figure 1g). Additionally, virtual gene panels were applied to 34 (23.1%) of the exome and genome sequencing studies.

**FIGURE 2:**
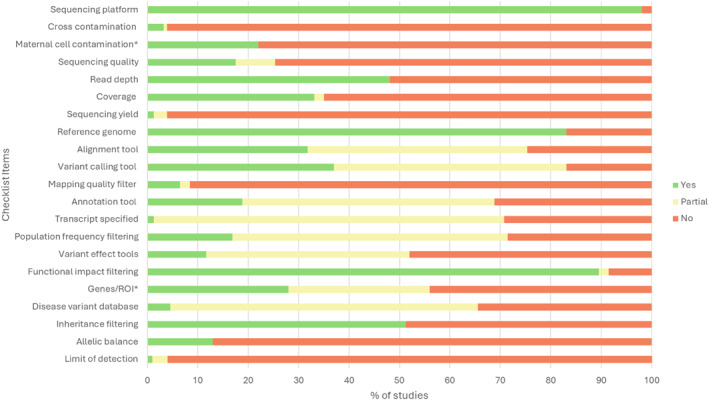
Quality assessment of bioinformatics details reported in included studies, expressed as a percentage of all studies. *n* = 154 for all items, except items labelled with *. ‘Maternal cell contamination’ and ‘Genes/ROI’ were applicable items in 151 and 60 studies, respectively. Studies were graded ‘Yes’ if the item was reported with details such as version number or date accessed for tools, or if cut‐offs were provided if a particular filter was applied. Studies were graded ‘Partial’ if some but not all components were reported and ‘No’ if the whole item was not reported.

Reporting of key bioinformatics pipeline details across the 154 studies is summarised in Figure 2. This figure provides an overview of the extent to which each parameter was fully, partially, or not reported, and serves as a central reference point for the analysis presented throughout the results section. To maintain clarity and avoid redundancy, Figure 2 will not be repeatedly cited in subsequent sections. However, readers are encouraged to refer to this figure as required.

### 3.2 Primary Analysis and Quality Control

The primary stage of bioinformatics analysis converts raw sequencer data to nucleotide bases. Illumina platforms were used in 89% (137/154) of studies, followed by MGI (15/154, 9.7%). One (0.6%) study used ION Torrent and three (1.9%) did not report the sequencing platform.

Assessing the quality of sequencing data is essential, as it directly affects the reliability of variant calls. Yet, quality control was rarely reported. Checks for cross‐contamination and maternal cell contamination were documented in just 4.5% (7/154) and 21.9% (33/151 prenatal samples) of studies, respectively.

**FIGURE 3:**
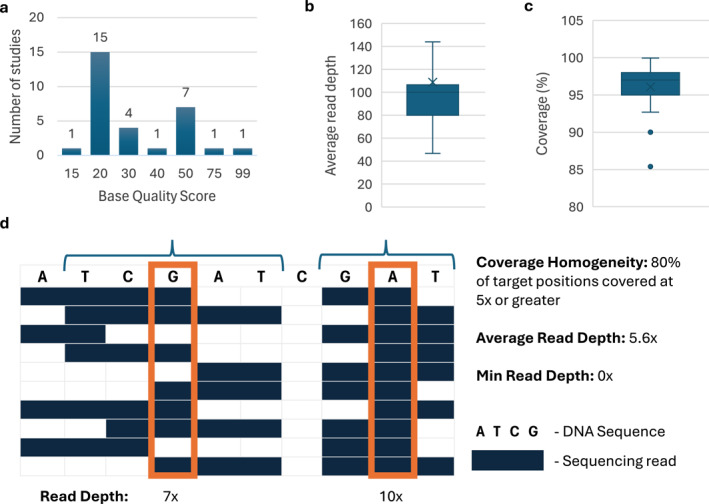
Quality metrics reported across 154 prenatal sequencing studies. (a) studies referencing Phred base quality scores (*n* = 30); (b) average read depth in exome sequencing studies (*n* = 77); (c) percentage of target region successfully sequenced across all studies (*n* = 54); (d) schematic illustrating read depth and coverage homogeneity.

Phred scores, indicating base call confidence, were reported in 19.5% (30/154) of studies, with representative scores ranging from 15 to 99 (Figure 3a). Average read depth was the most cited metric (74/154, 48.1%), followed by coverage (53/154, 34.4%), with both showing wide variability. Figure 3b illustrates the range of average depths across exome sequencing studies, while Figure 3c shows coverage across all studies.

While read depth and coverage are both essential quality metrics, these terms are frequently used interchangeably, requiring readers to infer their precise meaning from the study context. This lack of standardised terminology significantly hinders clear and consistent reporting. In this review, depth refers to the number of times a specific nucleotide position has been sequenced and is typically reported as a mean or median across all callable bases (e.g., average of 42.5x). Coverage describes the proportion of the target bases that have been successfully sequenced and is typically reported as a percentage (e.g., 95%). Coverage homogeneity integrates both metrics, offering a more comprehensive indication of data quality by reporting the proportion of target bases that meet a predefined sequencing depth (e.g., 95% of bases at ≥ 30x), as shown in Figure 3d. Only 33.8% (52/154) of the studies accurately reported both metrics.

Finally, yield provides a measure of the usable data post‐quality control and was only mentioned in 3.9% (6/154) of studies.

### 3.3 Secondary Analysis

The secondary stage of a bioinformatics pipeline is alignment of reads to a reference genome, followed by variant calling at discordant sites. Despite the release of GRCh38 in 2013, most studies used GRCh37 (120/154, 77.9%). Of the remainder, eight used GRCh38 (5.2%), while 26 (16.9%) did not specify the reference genome.

**FIGURE 4:**
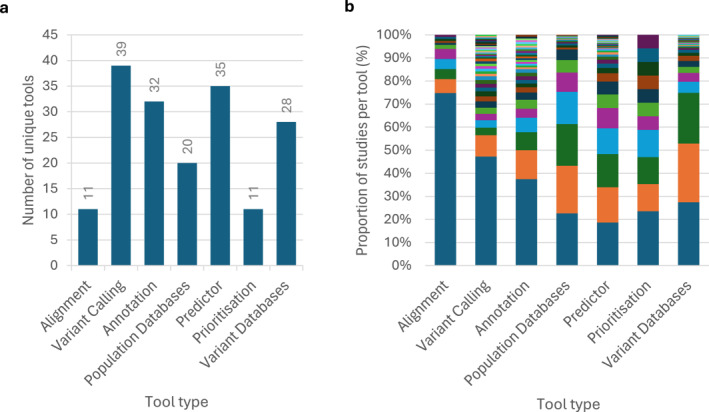
Bioinformatics tools used in 154 prenatal sequencing studies. (a) absolute number of each category of analysis tool used across studies; (b) proportion of studies utilising each tool.

Tool reporting was similarly inconsistent. The alignment and variant calling tools employed were omitted in 24.7% (38/154) and 16.2% (25/154) of studies, respectfully. Version details were reported less frequently. Of those that did report tools, 11 aligners and 53 variant callers were used (Figure 4a) with varying frequency (Figure 4b). One study also reported using GATK for alignment despite this not being an alignment tool.

Mapping quality (MQ) scores indicate confidence in read placement during alignment. Reads below a threshold may be filtered out. Thirteen studies (8.4%) reported MQ filtering, with MQ40 being the most common threshold (7/13, 53.8%). Three studies (23.1%) did not specify the threshold used.

### 3.4 Tertiary Analysis

The final stage of the bioinformatics pipeline involves annotating variants to provide a biological context, followed by filtering and prioritisation to identify those most likely to be disease‐causing.

### 3.5 Annotation Tool

Fifty studies (32.5%) did not specify the variant annotation tool used. Among those that did, 43 tools were recorded (Figure 4a–b).

### 3.6 Transcript Set

Transcripts represent various RNA isoforms from a gene and differ in exon composition. The choice of transcript can influence functional and clinical interpretation of the same genomic locus. While 69.5% of studies (107/154) included the reference transcript when reporting variants, only two (1.3%) specified which transcript sets were considered in methods.

### 3.7 Population Frequencies

**FIGURE 5:**
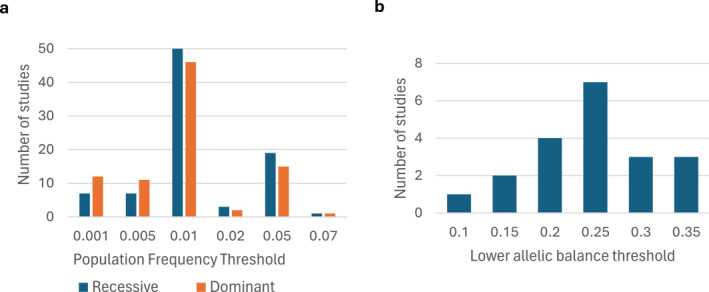
Variation in tertiary bioinformatics analysis parameters reported across 154 prenatal sequencing studies. (a) population frequency threshold applied for genes with an expected recessive and dominant inheritance pattern (*n* = 87); (b) the lower allelic balance threshold applied (*n* = 20).

A common strategy for filtering benign variants is to exclude those frequently observed in the population, using population databases and predefined allele frequency thresholds. The most used databases were 1000G (79/154, 51.3%) and gnomAD (72/154, 46.8%), with cut‐offs from < 0.001 to < 0.07 (Figure 5a). Multiple databases were often used, with 20 reported in total (Figure 4a–b). Forty‐four studies (28.6%) did not state whether a population database was used, although population filtering was evident in at least nine studies (20.5%). Of the 110 studies that reported using a population database, 32 (29.1%) omitted the frequency thresholds applied.

### 3.8 Variant Effect Predictors

Numerous variant effect predictor tools have been developed to try to predict the pathogenicity of variants, with 35 used across the reviewed studies (Figure 4a–b). These tools support variant classification by contributing to the overall evidence base. Of the 154 studies, 78 (50.6%) reported using at least one predictor tool, and 22 (28.2%) incorporated these tools into their filtering strategies. Specifically, five (22.7%) reported rescuing variants with scores indicative of pathogenicity, whereas three (13.6%) excluded variants predicted to be benign. The remaining 14 (63.6%) studies did not specify how filtering was applied.

### 3.9 Functional Impact

Variant annotation provides a biological context, including the predicted consequences. Broadly, filtering may focus on exonic regions or variants affecting protein‐coding sequences. More granularly, it can involve concentrating on functional impacts such as missense, frameshift or splice site changes. Of the 154 studies reviewed, 143 (92.9%) applied some form of functional filtering, though approaches and the level of methodological detail provided varied considerably.

### 3.10 Phenotype‐Based Filtering

There are two main approaches to variant filtering and prioritisation. Agnostic approaches are variant‐focused, assessing pathogenicity based solely on factors such as functional impact and population frequency without bias. The alternative is phenotype‐driven, which also considers the gene's relevance to clinical presentation, for example foetal structural abnormalities in this review.

This filtering can occur during primary analysis via targeted sequencing of phenotype‐associated gene panels. Alternatively, virtual panels can be applied to exome or genome sequencing data. Clinical exomes also restrict analysis to genes with established clinical relevance, though this is not phenotype driven. Of the 34 (22.1%) studies that used virtual gene panels, five (14.7%) reported switching to an agnostic approach if there was no pathogenic variant found. Overall, only 34/60 (56.7%) studies which reported using a clinical exome, panel capture, or a virtual panel reported the number of genes analysed, which ranged from 40 to 19,396.

Variant prioritisation tools combine genetic and phenotypic data to generate pathogenicity ‘scores’, which can be used to rank variants. Of the studies reviewed, 19 (12.3%) reported using such tools (Figure 4a–b) and five (26.3%) applied the scores for filtering or rescuing variants.

### 3.11 Disease‐Associated Variant Databases

Another common filtering strategy is leveraging existing knowledge. Disease‐associated variant databases provide a record of previously classified variants and evidence criteria based on the number of supporting and conflicting submissions. Laboratories may also curate in‐house databases, which were reported in nine (5.8%) studies. In total, 110/154 (71.4%) studies referenced one or more of 28 reported disease variant databases, most commonly ClinVar (85), HGMD (79) and OMIM (68) (Figure 4a–b). Among these, 14 (12.7%) reported rescuing or prioritising variants previously classified as (likely) pathogenic, with seven (50.0%) specifying the evidence criteria used for inclusion. Conversely, five (4.5%) studies used these databases to filter out variants previously classified as (likely) benign, but none reported applying evidence thresholds. Only seven (6.4%) studies recorded the database version or date accessed.

### 3.12 Inheritance Filtering

As aforementioned, 126/154 studies (81.8%) performed trio analysis when feasible (102 as trio‐only and 24 in combination with singleton analysis). This is a common practice in prenatal studies, and as such, inheritance filtering is often performed. This type of filtering excludes variants, which do not fit the expected inheritance pattern of the gene. Among the studies that conducted trio analysis, 41/102 (40.2%) reported performing inheritance‐based filtering, while 61 (59.8%) did not specify how the trio data was utilised.

### 3.13 Allelic Balance and Limit of Detection

For a given genotype, a 50:50 ratio of reads for each allele would be expected for a heterozygous variant, whereas nearly all reads should support the same allele for homozygous or X‐linked hemizygous variants. Thresholds are used to define the proportion of reads required to call a variant heterozygous or homozygous. For example, consider homozygous if > 80% of reads support one allele, whereas 30%–70% support for each allele indicates heterozygosity. Mosaic variants, which are genetic alterations present in only a subset of an individual's cells, can also have a low allelic balance percentage. Only 19 of the 154 (12.3%) studies provided information on allelic balance thresholds. Of these, 17 (89.5%) only provided information on the lower threshold, which varied from 10% to 35% (Figure 5b). Only one (0.6%) study reported the limit of detection for mosaic variants.

## 4 Discussion

The new review period yielded 1.35x more records than the previous [2], despite covering only half the duration, highlighting the growing volume of prenatal sequencing studies. Most genome sequencing also occurred during this recent period, reflecting a shift to more agnostic approaches. With continually increasing study numbers and data volume, standardised bioinformatic reporting is becoming essential.

### 4.1 Primary Analysis

Several studies failed to report the sequencing platform used, despite its relevance to data interpretation. Different platforms have varying error rates and suitability for detecting specific variant types [7], so platform choice directly influences confidence in variant calls and should be consistently reported.

Assessing the quality of sequencing data is essential to ensure its suitability for clinical analysis. However, few studies documented their quality control measures or thresholds applied to ensure data integrity. This lack of transparency makes it difficult to evaluate the robustness and reliability of the study design.

Approximately half of the studies did not report read depth or coverage, and those that did often confused or used the terms interchangeably. Read depth is the number of sequence reads covering a base, which informs confidence in variant calls and allelic balance. Conversely, coverage describes the proportion of target regions sequenced, which is crucial for identifying gaps. Both metrics should be reported to provide a comprehensive picture of data quality, i.e. the percentage of target bases that achieve a predefined sequencing depth (e.g., 95% at ≥ 30x). Additionally, any clinically significant targets in difficult‐to‐sequence regions (e.g., low complexity or highly repetitive) should be explicitly assessed and reported.

### 4.2 Secondary Analysis

Many studies failed to report the reference genome as well as the tools and their versions used for alignment and variant calling. However, this information is essential for reproducibility and understanding potential limitations.

For example, among studies that specified a reference genome, most were still using GRCh37. This older build provides a less complete picture of the genome compared to GRCh38 and offers poorer representation of repetitive regions, including no coverage of centromeres and telomeres. Studies have also shown that GRCh38 enables more accurate genomic analysis, particularly for structural variants [8].

Similarly, alignment and variant calling tools, and their specific versions, can introduce tool‐specific biases. Without this information, reliability cannot be assessed. A benchmarking study of nine variant callers demonstrated that tool selection significantly affects accuracy, with wide variation in sensitivity and susceptibility to factors like depth and GC content [9]. These differences have clinical implications, as a pathogenic variant could be missed by one tool but detected by another.

### 4.3 Tertiary Analysis

As with secondary analysis, many studies did not document the tool(s) used for variant annotation. Similarly, the transcript sets considered were omitted in nearly all cases. Both are crucial, as they directly influence the predicted molecular consequences of potentially pathogenic variants and, in turn, whether they are included or excluded from further interpretation. Notably, a recent study revealed significantly different transcript expression patterns between prenatal and postnatal cortex samples. This included thousands of novel prenatal transcripts with high coding potential, highlighting untapped coding sequences where pathogenic variants may reside [10].

Many studies did not adequately report the population databases or allele frequency thresholds used during variant filtering. This information is critical, as population frequency is often applied as a hard filter that determines which variants are retained for interpretation. Consequently, potentially pathogenic variants, particularly those with low penetrance or variable expressivity, may be inadvertently filtered out due to their higher prevalence in the population. This is especially relevant in prenatal settings, where some phenotypes manifest more severely than postnatally, so causative variants may exceed allele frequency thresholds [11, 12, 13]. Hypomorphic variants, which partially reduce gene function, can also surpass frequency thresholds. Several such variants have been associated with thrombocytopaenia–absent radius syndrome [11, 12, 13, 14]. Additionally, allele frequencies vary across ethnic groups, making it essential to understand whether ancestry and population diversity have been considered in the study design. Without this, results cannot be interpreted within the context of their potential limitations.

Variant effect predictors are valuable aids, especially as data volume increases with exome and genome sequencing. While many reviewed studies mentioned annotating variants using these tools, only 22 explicitly reported incorporating them into a filtering strategy. Of these, over half lacked detailed methodology.

Although such tools can offer useful insights, their accuracy in predicting variant pathogenicity is limited. Performance varies by variant type, functional impact, and biological context, as previously reviewed [12]. Hard filtering based on these predictions should be applied cautiously and ideally only in line with specific recommendations. Conversely, when using predictions to filter in potentially pathogenic variants, such as splice variants using SpliceAI, it is essential to document applied thresholds to fully understand the scope of the findings.

Most reviewed studies applied some form of functional filtering, but strategies and methodological details varied considerably. Transparent reporting is essential for accurate interpretation; otherwise, readers may assume that all variant types were considered when pathogenic variants may have been excluded. This is especially relevant given the growing recognition of non‐coding variants as important contributors to genetic diseases [13].

Significant numbers of studies reported limiting their analysis to panels of genes known to be associated with specific prenatal structural abnormalities, and approximately 10% utilised a tool with built‐in phenotypic prioritisation. While valid, this approach excludes genes with limited or no known disease‐gene association. However, this is a continually evolving field. Moreover, phenotypes may be less well‐defined prenatally than postnatally, which will impact the success of this method [14]. As such, reporting the included genes and the version of the prioritisation tool or the date it was accessed provides an essential context for interpretation.

One way to minimise the loss of pathogenic variants when applying hard filters is to ‘rescue’ variants previously classified as pathogenic in public or in‐house disease‐associated variant databases. For example, one study reported that ‘variants with a minor allele frequency (MAF) > 5% were filtered out except for those in HGMD, ClinVar and ClinGen B1 exception list (BA1)’ [15]. This approach retains known pathogenic variants with low penetrance that would otherwise be excluded in frequency‐based filtering. However, its application in prenatal settings has limitations due to previously mentioned differences in the severity of phenotypes [11, 12, 13, 16, 17, 18].

Approximately two‐thirds of studies reported annotating with disease‐variant databases, but only 14 explicitly used them to rescue variants, and just seven recorded the version or date accessed. This is particularly important for disease‐variant databases as they are frequently updated, with ClinVar releasing new classifications every month. Therefore, a variant not rescued 1 month may be the following due to an updated classification, making date‐tracking essential to fully understand the scope of findings. Incidental findings and variants with no clear phenotype pose additional challenges, often leading to uncertain results. To mitigate this, many studies adopt restricted approaches. However, without clear documentation of database versions and filtering strategies, it becomes difficult to assess the scope and limitations of the analysis.

Six studies reported using disease‐variant databases to filter out variants previously classified as benign. This method requires caution, as new evidence can lead to reclassification, potentially resulting in the erroneous exclusion of pathogenic variants. One way to mitigate this risk is to apply evidence‐based thresholds, such as only excluding variants with multiple submissions and no conflicting interpretations. However, none of these studies reported using such criteria. Again, documenting this information is therefore critical to understanding limitations in results.

Inheritance filtering can be valuable for identifying disease‐causing variants in prenatal studies. De novo variants are the most common cause of postnatal genetic disease, and 47% of foetuses undergoing exome sequencing for structural abnormalities are expected to have such variants [19]. Trio testing can identify de novo variants and clarify whether two variants in the same gene with expected autosomal recessive inheritance are inherited from a different parent (i.e., in trans). At least 40% of the studies in this review applied inheritance filtering to exclude variants inconsistent with the expected inheritance pattern of the gene. However, inheritance filtering can be complex in prenatal analysis for several reasons, including some prenatal phenotypes being more pronounced than postnatal ones [16, 17, 18], and lethal variants potentially exhibiting inheritance patterns that differ from those known for a given gene [20]. Therefore, reporting whether inheritance filtering has been applied is essential, so these factors can be considered in the interpretation of findings.

Finally, fewer than 15% of the reviewed studies provided allelic balance thresholds. If too stringent, pathogenic variants may be missed. Conversely, if too lenient, homozygous variants could be miscalled as heterozygous, which can affect diagnosis, especially in recessive genes. Reduced specificity may also occur due to sequencing noise being misinterpreted as variants, risking misdiagnosis. Moreover, it is important to understand the scope of the test. For example, if excluding variants with an allelic balance of less than 20%, pathogenic mosaic variants could be missed.

### 4.4 Strengths and Limitations

This is the first systematic review to assess bioinformatic pipelines in prenatal sequencing, providing a comprehensive framework for future reporting. It underscores the clinical importance of transparent methodology and details key elements that should be documented. However, the completeness of reporting may be limited by journal word restrictions and, in some cases, by proprietary commercial software that may not disclose essential protocol details.

## 5 Conclusion

The rise in prenatal sequencing studies reflects growing interest and recognition of its clinical utility. Combined with the shift toward exome and genome sequencing, this underscores the need for robust bioinformatic analysis strategies.

This review does not aim to provide prescriptive guidelines on bioinformatics analysis, as approaches should be tailored to the specific requirements of each study or service. Instead, it highlights how analytical decisions can have direct clinical consequences, particularly in the prenatal setting. We identified deficiencies in current reporting practices and illustrated how these gaps prevent full interpretation of the results and understanding of study limitations. Based on our findings and to aid interpretation of sequencing studies, we propose a checklist in Table 1 that can be used for reporting bioinformatic strategies.

**TABLE 1:** Recommendations for reporting bioinformatics methodologies.

Metric	Recommendation
Sequencing platform	Specify platform and model.
Coverage homogeneity	State read depth and coverage across target regions (e.g., 95% > 30x)
Sequencing quality	Report number of on‐target bases per sample post quality control that exceed a predefined Phred score (e.g., 85Gb > Q30)
Contamination check	Report if screened for cross/maternal cell contamination; confirm contamination < set threshold (e.g., 3%)
Reference genome	Specify reference genome used for alignment (e.g., GRCh38)
Tools	Provide tool names, versions, and/or date accessed (e.g., BWA v0.7.19)
Mapping quality	State minimum mapping quality if applied (e.g., < MQ40 removed)
Transcript(s)	Specify transcript resource(s) used for annotation (e.g., MANE plus clinical)
Population frequency	State allele frequency thresholds applied for filtering (e.g., exclude variants with a minor allele frequency > 0.01 in gnomAD v4)
Variant effect predictors	State in silico tool thresholds for filtering/rescuing variants (e.g. rescue if CADD > 20)
Functional filters	State functional filtering parameters (e.g. only non‐synonymous, splice site, nonsense & frameshift variants analysed)
Phenotype‐based filters	State phenotype‐based filters, including prioritisation tools and parameters; if a gene panel was used, provide name, version, and/or date accessed (e.g. PanelApp fetal anomalies v6.93) or the gene list.
Variant databases	State criteria for rescuing/filtering variants using external databases/tools; specify evidence requirements (e.g., include variants reported as pathogenic in HGMDv2025.2, no minimum evidence required).
Inheritance‐based filters	State inheritance‐based filters (e.g. exclude variants not matching expected segregation pattern)
Allelic balance	State allelic balance thresholds for homozygous (e.g., > 80%) and heterozygous (e.g., 35%–65%) variants
Limit of detection	State limit of detection (e.g., mosaic variants could be detected down to a variant allele fraction of 5%).

It is recommended that authors incorporate this checklist during manuscript preparation, and that journals consider adopting it within their submission guidelines. Such implementation would help to ensure that the details of bioinformatic methodologies are consistently reported, thereby supporting reproducibility and enabling meaningful comparison across studies.

As genomic technologies continue to advance, this framework must adapt to incorporate changing and emerging practices. For example, long‐read sequencing introduces new parameters such as read‐length distribution and phasing accuracy. We hope this review prompts authors to carefully consider which metrics are essential for comprehensive and transparent reporting as the field evolves.

To conclude, this review highlights the necessity for comprehensive and transparent bioinformatic reporting to ensure data integrity and reliability, and to support interpretation of the literature. If this standardisation of bioinformatic reporting can be implemented, it will aid reproducibility, allowing further expansion of knowledge of prenatal sequencing.

## Funding

A.J.P. and E.S. are funded by the NIHR Biomedical Research Center at Great Ormond Street Hospital. G.S. and A.G. are funded by the North Thames Genomic Medicine Service Alliance. K.L. is funded by the Ministry of Health Singapore.

## Ethics Statement

The authors have nothing to report.

## Consent

The authors have nothing to report.

## Conflicts of Interest

The authors declare no conflicts of interest.

## Data Availability

The data that support the findings of this study are available from the corresponding author upon reasonable request.
